# Global, regional, and national burden of neonatal otitis media attributable to PM2.5 air pollution: findings from the Global Burden of Disease study 2021

**DOI:** 10.3389/fpubh.2025.1625071

**Published:** 2025-12-04

**Authors:** Xin Gong, Yinxia Xu, Yuxi Tu, Chao Yuan, Zhenhong Zheng, Faling Song, Mengyu Rao, Yan Liu, Deping Wang

**Affiliations:** 1 Department of Otolaryngology, Wushan County People’s Hospital of Chongqing, Chongqing, China; 2 Department of Paediatrics, Wushan County People’s Hospital of Chongqing, Chongqing, China; 3 Department of Otolaryngology, The First Affiliated Hospital of Chongqing Medical University, Chongqing, China; 4 Department of Radiology, Wushan County People’s Hospital of Chongqing, Chongqing, China; 5 Department of Ultrasonography, Wushan County People’s Hospital of Chongqing, Chongqing, China; 6 Department of Otolaryngology, Kaizhou District People’s Hospital of Chongqing, Chongqing, China; 7 Department of Otolaryngology, The Second Affiliated Hospital of Chongqing Medical University, Chongqing, China

**Keywords:** otitis media, PM2.5, air pollution, neonates, disease burden, GBD

## Abstract

**Background:**

Long-term exposure to fine particulate matter (PM2.5) may increase the risk of otitis media (OM) in neonates. However, the global burden of neonatal OM attributable to PM2.5 remains poorly understood. This study aims to assess the burden and epidemiological trends in neonatal OM (0–28 days old) attributable to PM2.5 from 1990 to 2021.

**Methods:**

Using data from the Global Burden of Disease (GBD) study 2021, we estimated the number and rates of disability-adjusted life years (DALYs) and years lived with disability (YLDs) associated with PM2.5-related OM in neonates. This analysis was stratified by sex, sociodemographic index (SDI), region, and country. The estimated annual percentage change (EAPC) method was used to assess temporal trends. Decomposition analysis was conducted to identify the main drivers of change.

**Results:**

Globally, the burden of neonatal OM attributable to PM2.5 levels declined from 1,363.136 DALYs (95% uncertainty interval [UI]: 512.175–2,946.844) in 1990 to 327.396 DALYs (95% UI: 156.269–626.363) in 2021. YLDs also decreased from 178.925 (95% UI: 82.305–314.825) to 165.301 (95% UI: 75.609–314.563). The burden of neonatal OM attributable to PM2.5 levels varied substantially across countries and regions, with higher numbers and rates of DALYs and YLDs in less-developed areas, where household PM2.5 pollution continues to play a crucial role. Decomposition analysis showed that epidemiological changes were the primary drivers behind the global decline in DALYs and YLDs, while population aging contributed to the increasing burden in low SDI regions.

**Conclusion:**

Although the global burden of neonatal OM attributable to PM2.5 exposure has declined over the past three decades, it remains a persistent issue, particularly in resource-limited settings. Targeted interventions to reduce PM2.5 exposure—especially from household sources—are urgently needed to address these health disparities.

## 1 Introduction

Ambient air pollution, particularly particulate matter (PM), has emerged as a critical public health challenge with significant global impacts, contributing to millions of deaths and disability-adjusted life years (DALYs) annually (1–3). PM2.5, classified as a Group 1 carcinogen by the International Agency for Research on Cancer, is one of the major drivers of both health burdens and economic losses (4, 5). Recent estimates suggest that, in 2019, PM2.5 contributed to 4.14 million deaths and 118.2 million DALYs globally (6). These fine particles can deeply penetrate the respiratory system, posing health risks across all ages, with particularly severe impacts on fetuses and neonates (7).

Otitis media (OM), a common inflammatory disease of the middle ear, is a significant health concern for children worldwide, with acute otitis media being the second most common childhood illness after upper respiratory tract infections (8, 9). OM can lead to hearing loss, developmental delays, and other adverse outcomes (10). Neonates, with their underdeveloped immune systems, are particularly vulnerable to OM, which often occurs as a complication of acute upper respiratory tract infections (11, 12). Previous studies have demonstrated a significant association between PM2.5 exposure and an increased risk of OM, including neonatal infections and other adverse neonatal outcomes (13, 14).

Despite the growing body of evidence linking PM2.5 exposure to OM, no comprehensive assessment has examined the burden of neonatal OM attributable to PM2.5. Understanding the epidemiological trends and disease burden of neonatal OM linked to PM2.5 is crucial for policymakers to allocate health resources effectively and develop targeted prevention strategies. Therefore, this study aims to analyze the global, regional, and national trends in the burden of neonatal OM attributable to PM2.5 from 1990 to 2021, based on the Global Burden of Disease (GBD) study 2021. Our findings are intended to inform evidence-based strategies for effective prevention and treatment policies.

## 2 Methods

### 2.1 Data extraction and case definition

This study utilized data from the GBD study 2021^1^ to analyze the epidemiological trends and the disease burden of OM attributable to PM2.5 in neonates (0–28 days) globally. The GBD project systematically assesses the burden of 371 diseases and injuries, along with 88 risk factors, across 204 countries and territories (15). In the GBD database, OM is defined as an infection of the middle ear space and is categorized under ICD-10 codes H65–H75. PM2.5 pollution is classified as a Level 3 risk factor, including both ambient PM2.5 and household PM2.5 concentrations. Ambient PM2.5 is measured as the population-weighted annual average concentration of fine particulate matter per cubic meter of air, while household PM2.5 is primarily generated by burning solid fuels for cooking (16). Further details regarding the modeling techniques employed can be found on the Institute for Health Metrics and Evaluation (IHME) website^2^.

In the GBD study 2021, the relationship between risk factors and health outcomes was evaluated using a comparative risk assessment framework. Briefly, exposure levels were estimated across populations; their associations with disease outcomes were quantified based on pooled relative risks from published studies; and the potential impact of reducing exposures to an ideal reference level was then calculated. Statistical adjustments were applied to ensure that overlapping risks were not counted more than once. It should also be noted that the attribution does not imply that each case of OM is directly caused by PM2.5 exposure; instead, it reflects the percentage of the disease burden that could be avoided at the population level if exposure to PM-related pollutants were reduced (17).

We extracted the number and rate of disability-adjusted life years (DALYs) and years lived with disability (YLDs) for neonatal OM burden attributable to PM2.5, along with their 95% uncertainty intervals (UIs), stratified by year, sex, region, and country. DALYs, a composite indicator of overall disease burden, are the sum of years of life lost (YLLs) and YLDs. The 204 countries and territories were divided into 21 GBD regions based on geographical locations. The sociodemographic index (SDI, data source: https://ghdx.healthdata.org/gbd-2021) was used to stratify countries into five groups as follows: low, low-middle, middle, high-middle, and high SDI regions (18).

### 2.2 Statistical analysis

We assessed regional and national differences using DALY and YLD rates. The estimated annual percentage change (EAPC) method was used to characterize temporal trends in OM attributable to PM2.5. A log-linear regression model was fitted to the natural logarithm of the DALY or YLD rate over time, with the EAPC calculated as 100 × (exp(*β*) − 1), where β is the slope of the regression line. An upward trend was indicated by an EAPC with a lower 95% confidence interval (CI) greater than 0, while a downward trend was indicated by an EAPC with an upper 95% CI less than 0.

Hierarchical cluster analysis was performed to identify regions with similar changes in the disease burden. Decomposition analysis is a statistical approach that breaks down an overall change into the contributions of various factors, aiming to identify the factors that exert a significant impact on the change and quantify the extent of their influence (19). We utilized the Das Gupta decomposition method to quantify the contributions of population growth, aging, and epidemiological changes to the overall disease burden of neonatal OM attributable to PM2.5 from 1990 to 2021. *p*-values less than 0.05 were considered statistically significant. All analyses were conducted using R software (version 4.4.2).

## 3 Results

### 3.1 Spatiotemporal patterns of neonatal OM burden attributable to PM2.5

From 1990 to 2021, global DALYs and YLDs due to neonatal OM attributable to PM2.5 decreased significantly. DALYs declined from 1,363.14 (95% UI: 512.18–2,946.84) in 1990 to 327.396 (95% UI: 156.269–626.363) in 2021, representing a 76.0% reduction (Table 1). YLDs decreased from 178.93 (95% UI: 82.31–314.83) in 1990 to 165.30 (95% UI: 75.61–314.56) in 2021, corresponding to a 7.6% reduction (Supplementary Table S1). The DALY rate decreased from 0.220 per 100,000 in 1990 to 0.050 in 2021, indicating a 77.3% decrease. The EAPC for the DALY and YLD rates was −4.782 (95% CI: −5.210 to −4.352) and −0.493 (95% CI: −0.572 to −0.413), respectively.

**Table 1:** DALY cases and rates for neonatal OM attributable to PM2.5 in 1990 and 2021, and the EAPC from 1990 to 2021 at the global and regional levels.

Location	1990		2021		1990–2021
	DALY cases, (95% UI)	DALY rate, per 100, 000 (95% UI)	DALY cases, (95% UI)	DALY rate, per 100, 000 (95% UI)	EAPC, %, (95% CI)
Global	1363.136 (512.175, 2946.844)	0.220 (0.083, 0.475)	327.396 (156.269, 626.363)	0.050 (0.024, 0.095)	−4.782 (−5.210, −4.352)
SDI					
High	33.247 (21.599, 44.032)	0.054 (0.035, 0.071)	4.048 (2.125, 7.034)	0.008 (0.004, 0.013)	−5.019 (−5.627, −4.407)
High-middle	43.537 (28.160, 62.612)	0.047 (0.030, 0.067)	6.227 (3.133, 11.331)	0.009 (0.004, 0.016)	−4.493 (−4.811, −4.173)
Middle	160.241 (99.153, 235.522)	0.080 (0.049, 0.117)	32.023 (16.915, 58.052)	0.018 (0.010, 0.033)	−3.827 (−4.202, −3.450)
Low-middle	523.212 (93.478, 1586.695)	0.302 (0.054, 0.915)	71.834 (34.692, 133.927)	0.037 (0.018, 0.070)	−5.856 (−6.657, −5.048)
Low	601.827 (223.230, 1402.169)	0.663 (0.246, 1.544)	213.006 (89.491, 472.662)	0.129 (0.054, 0.285)	−5.558 (−6.152, −4.960)
Regions					
Andean Latin America	3.647 (1.246, 9.309)	0.069 (0.024, 0.176)	1.134 (0.514, 2.257)	0.018 (0.008, 0.037)	−4.622 (−4.802, −4.442)
Australasia	0.101 (0.008, 0.315)	0.007 (0.001, 0.020)	0.075 (0.009, 0.204)	0.004 (0.001, 0.011)	−0.775 (−1.107, −0.441)
Caribbean	1.621 (0.566, 4.410)	0.039 (0.014, 0.107)	1.293 (0.495, 3.520)	0.033 (0.013, 0.091)	−0.530 (−0.753, −0.306)
Central Asia	1.459 (0.827, 2.525)	0.015 (0.009, 0.027)	0.816 (0.386, 1.466)	0.008 (0.004, 0.015)	−2.173 (−2.688, −1.655)
Central Europe	38.948 (24.068, 57.814)	0.427 (0.264, 0.633)	0.491 (0.218, 0.956)	0.009 (0.004, 0.017)	−11.051 (−12.361, −9.721)
Central Latin America	30.088 (19.875, 41.816)	0.131 (0.086, 0.182)	2.604 (1.316, 4.703)	0.013 (0.007, 0.023)	−6.708 (−7.344, −6.067)
Central sub-Saharan Africa	28.005 (4.163, 95.851)	0.270 (0.040, 0.923)	12.346 (3.727, 40.632)	0.059 (0.018, 0.193)	−5.102 (−5.284, −4.919)
East Asia	40.269 (19.745, 69.221)	0.035 (0.017, 0.060)	7.640 (3.280, 14.938)	0.010 (0.004, 0.019)	−3.068 (−3.307, −2.828)
Eastern Europe	1.873 (0.967, 3.342)	0.011 (0.006, 0.019)	0.288 (0.134, 0.542)	0.003 (0.001, 0.005)	−4.258 (−4.662, −3.853)
Eastern sub-Saharan Africa	461.615 (205.006, 1061.966)	1.279 (0.568, 2.943)	171.137 (64.810, 409.268)	0.268 (0.102, 0.642)	−5.250 (−6.095, −4.398)
High-income Asia Pacific	0.974 (0.475, 1.824)	0.010 (0.005, 0.018)	0.444 (0.176, 0.916)	0.007 (0.003, 0.014)	−1.057 (−1.311, −0.803)
High-income North America	2.949 (1.815, 4.625)	0.014 (0.008, 0.021)	0.853 (0.446, 1.492)	0.004 (0.002, 0.007)	−3.610 (−3.799, −3.421)
North Africa and the Middle East	10.428 (4.889, 20.367)	0.020 (0.010, 0.040)	12.958 (5.851, 24.455)	0.021 (0.010, 0.040)	0.186 (0.067, 0.304)
Oceania	0.342 (0.146, 0.656)	0.034 (0.014, 0.065)	0.647 (0.275, 1.247)	0.033 (0.014, 0.064)	0.001 (−0.033, 0.034)
South Asia	611.287 (74.998, 1995.559)	0.389 (0.048, 1.271)	62.489 (28.997, 118.707)	0.039 (0.018, 0.075)	−6.333 (−7.442, −5.212)
Southeast Asia	16.266 (7.816, 31.816)	0.028 (0.013, 0.055)	9.981 (4.504, 18.958)	0.018 (0.008, 0.034)	−1.532 (−1.616, −1.447)
Southern Latin America	0.867 (0.445, 1.525)	0.017 (0.009, 0.030)	0.287 (0.103, 0.632)	0.007 (0.002, 0.015)	−3.968 (−4.353, −3.581)
Southern sub-Saharan Africa	2.691 (1.194, 5.151)	0.036 (0.016, 0.069)	1.934 (0.812, 3.717)	0.024 (0.010, 0.046)	−1.123 (−1.355, −0.889)
Tropical Latin America	77.208 (48.489, 109.616)	0.452 (0.284, 0.642)	6.306 (3.607, 9.806)	0.037 (0.021, 0.057)	−5.497 (−6.528, −4.455)
Western Europe	16.334 (10.359, 22.728)	0.071 (0.045, 0.099)	2.190 (1.208, 3.767)	0.010 (0.006, 0.018)	−5.567 (−6.072, −5.058)
Western sub-Saharan Africa	16.164 (7.685, 30.010)	0.045 (0.022, 0.084)	31.482 (14.719, 59.569)	0.039 (0.018, 0.075)	−0.536 (−0.587, −0.485)

DALYs, disability-adjusted life years; OM, otitis media; PM2.5, fine particulate matter; EAPC, estimated annual percentage change; SDI, socio-demographic index; UI, uncertainty intervals; CI, confidence intervals.

**Figure 1:**
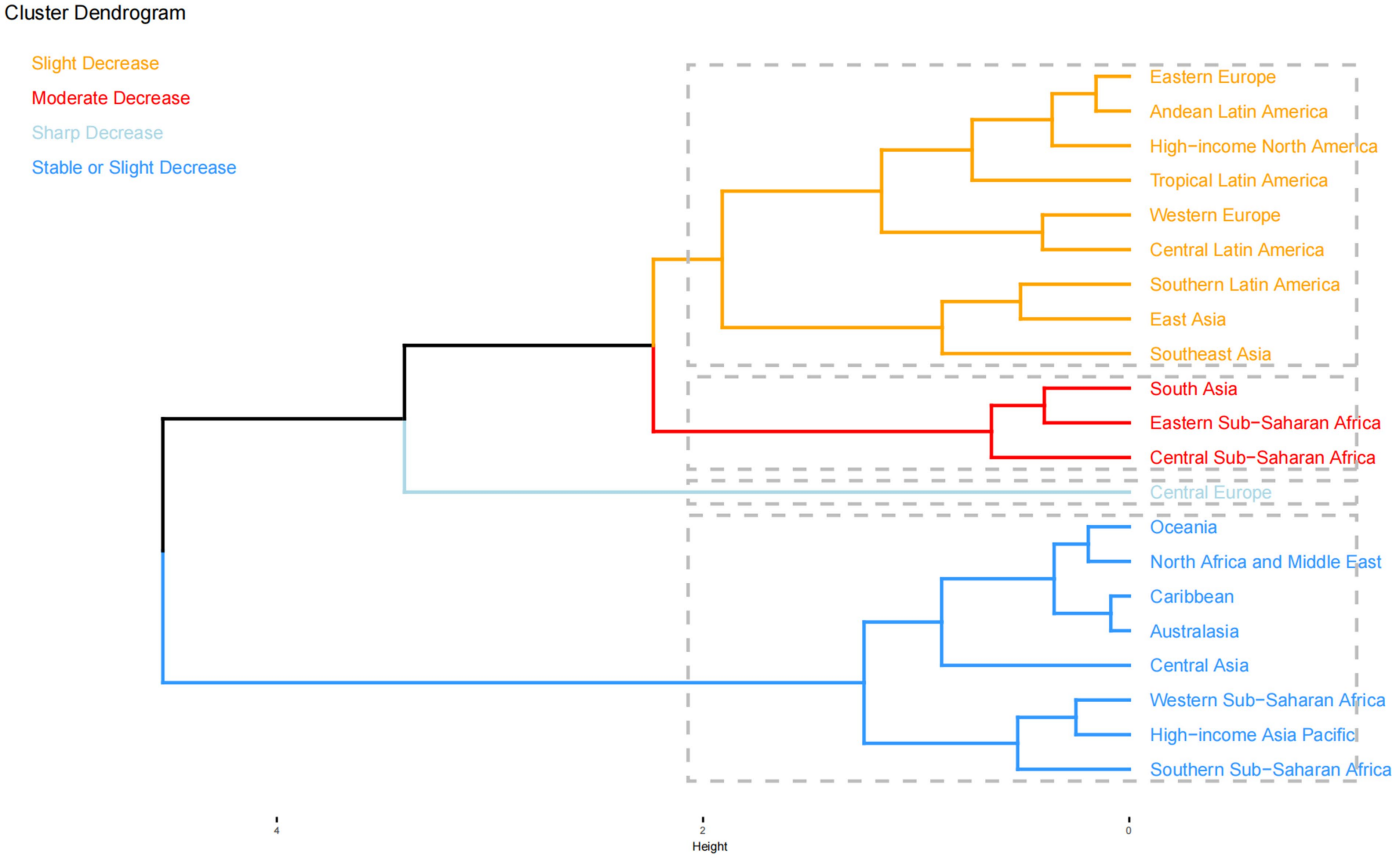
Results of hierarchical clustering analysis based on the EAPC values of neonatal OM attributable to PM2.5 for DALY and YLD rates from 1990 to 2021. EAPC, estimated annual percentage change; OM, otitis media; PM2.5, fine particulate matter; DALYs, disability-adjusted life years; YLDs, years lived with disability.

Regional DALY and YLD rates showed varied trends, with significant declines in some regions and more moderate decreases in other regions. Central Europe experienced the largest DALY rate decrease (EAPC: −11.051), while tropical Latin America saw the largest decline in the YLD rate. In 2021, Eastern sub-Saharan Africa had the highest DALY rate (171.137, 95% UI: 64.810–409.268), followed by South Asia and Western sub-Saharan Africa. Hierarchical clustering analysis (Figure 1) revealed distinct regional clusters with similar patterns of disease burden change, highlighting sharp decreases in Central Europe and moderate declines in South Asia and sub-Saharan Africa. Other regions exhibited stable or slightly decreased DALY and YLD rates.

**Figure 2:**
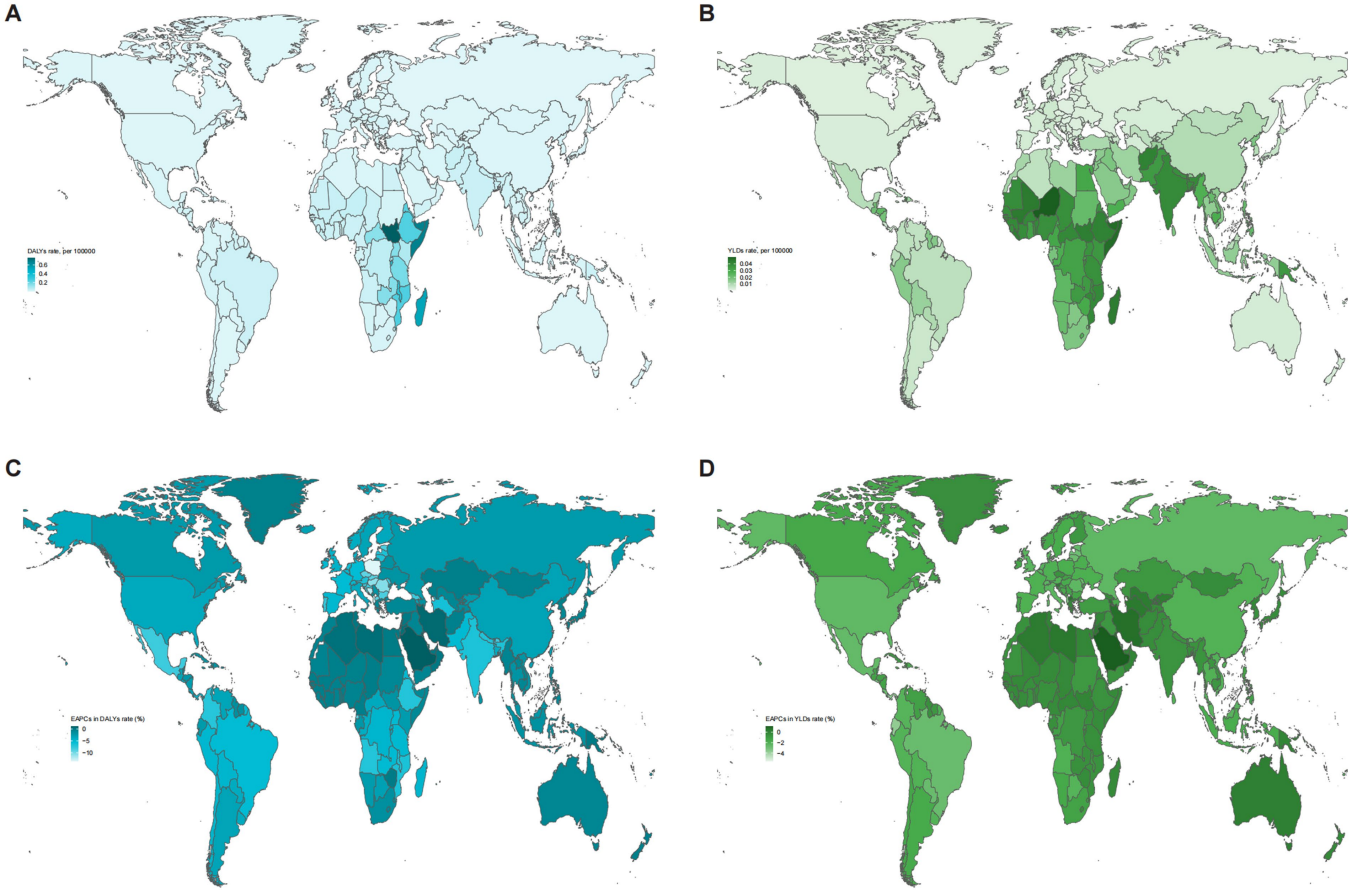
Global distribution (2021) and temporal trends (1990–2021) in DALY and YLD rates of neonatal OM attributable to PM2.5 across 204 countries and territories: **(A)** DALY rates; **(B)** YLD rates; **(C)** EAPC of DALY rates; **(D)** EAPC of YLD rates. DALYs, disability-adjusted life years; YLDs, years lived with disability; OM, otitis media; PM2.5, fine particulate matter; EAPC, estimated annual percentage change.

The global distribution and temporal trends in DALY and YLD rates for neonatal OM attributable to PM2.5 across the 204 countries and territories are shown in Figure 2. At the national level, 12 countries—mostly in North Africa and the Middle East—still showed an upward trend in DALY rates, with Saudi Arabia and Poland showing the largest increases and decreases, respectively. Meanwhile, South Sudan had the highest DALY rate in 2021 (0.754, 95% UI: 0.208–2.087), while Slovenia had the lowest rate (0.002, 95% UI: 0.000–0.005).

**Figure 3:**
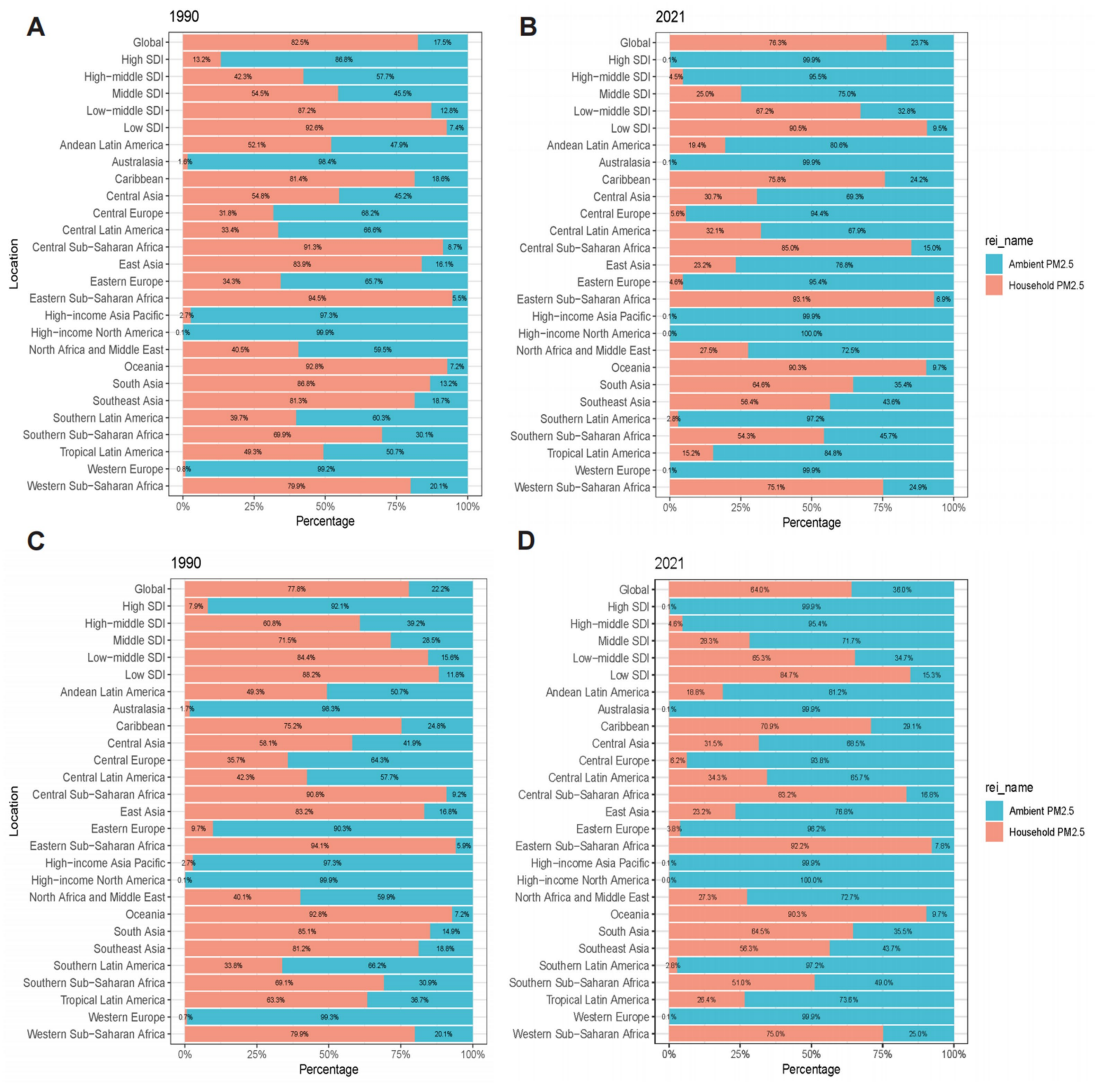
Contribution of ambient PM2.5 and household PM2.5 to the PM2.5-attributable neonatal OM burden across different locations: **(A)** DALYs in 1990; **(B)** DALYs in 2021; **(C)** YLDs in 1990; **(D)** YLDs in 2021. PM2.5, fine particulate matter; OM, otitis media; DALYs, disability-adjusted life years; YLDs, years lived with disability; SDI, socio-demographic index.

Examining the contributions of ambient and household PM2.5 to the burden of OM attributable to PM2.5, household PM2.5 accounted for 76.3% of DALYs and YLDs globally in 2021. Ambient PM2.5 contributed more significantly to the burden in high- and high-middle SDI regions, while household PM2.5 was more pronounced in low-middle and low SDI regions (Figure 3). High-income regions, such as North America, Asia Pacific, Western Europe, and Australasia, had higher DALYs and YLDs from ambient PM2.5, whereas less developed regions, including Eastern sub-Saharan Africa and Oceania, had a greater burden from household PM2.5.

### 3.2 Burden and trends by sex

**Figure 4:**
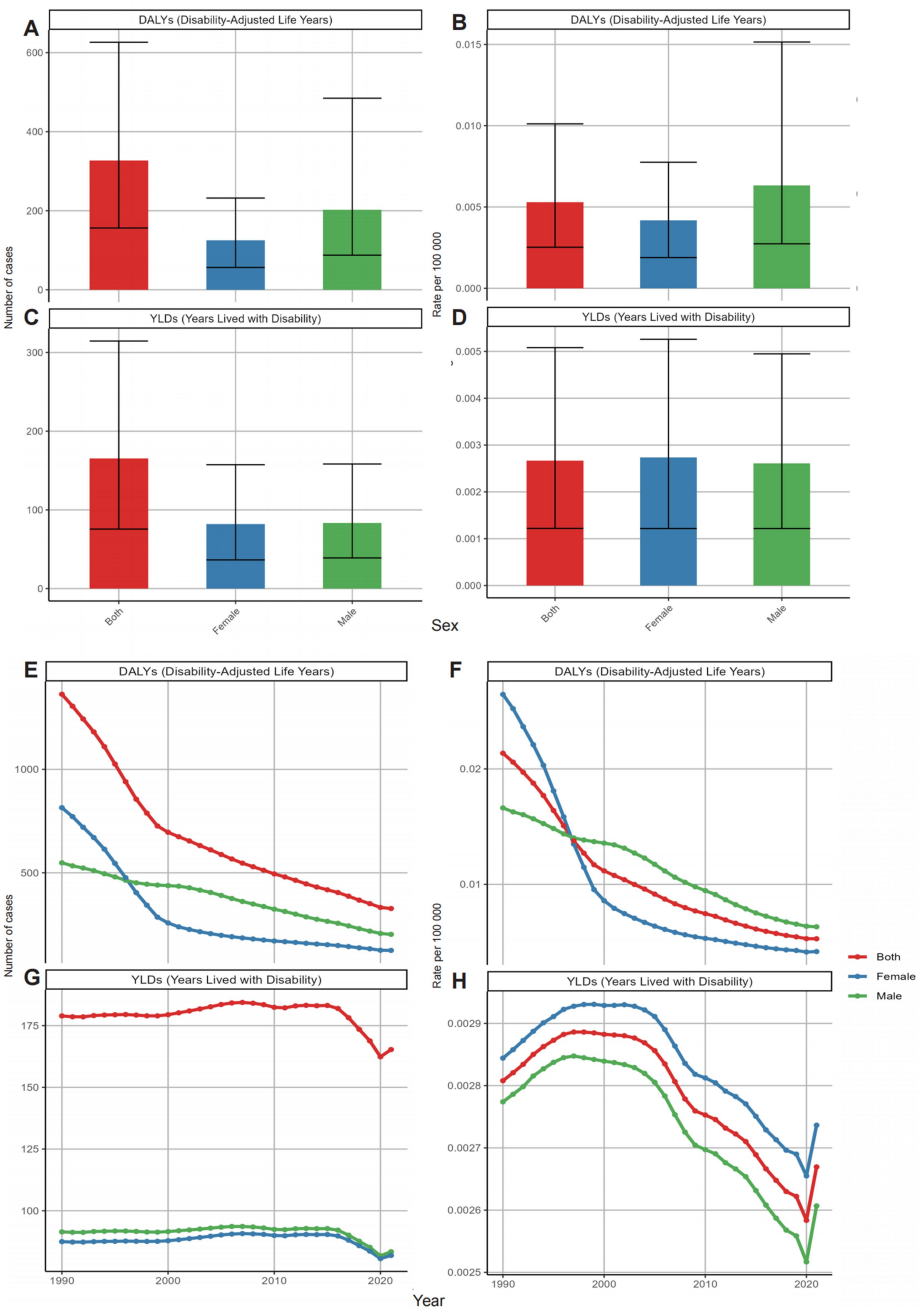
Global distribution (2021) and temporal trends (1990–2021) in neonatal OM attributable to PM2.5 by sex: **(A)** DALY cases; **(B)** DALY rates; **(C)** YLD cases; **(D)** YLD rates; **(E)** trends in DALY cases; **(F)** trends in DALY rates; **(G)** trends in YLD cases; **(H)** trends in YLD rates. DALYs, disability-adjusted life years; YLDs, years lived with disability; OM, otitis media; PM2.5, fine particulate matter.

In 2021, male individuals exhibited higher DALY cases and rates globally compared to female individuals, with DALYs totaling 202.408 (95% UI: 87.580–464.706) for male individuals and 124.988 (95% UI: 56.509–232.048) for female individuals. DALY rates were 0.060 per 100,000 for male individuals and 0.039 per 100,000 for female individuals (Figure 4). Conversely, YLD cases and rates were comparable between the sexes. Interestingly, before 1997, female individuals had higher DALY cases and rates than male individuals. From 1990 to 2021, DALY cases and rates declined in both sexes. However, despite the overall downward trend during the study period, YLD cases and rates showed slight increases in both sexes after 2020 (Figure 4).

### 3.3 Burden and trends by SDI

**Figure 5:**
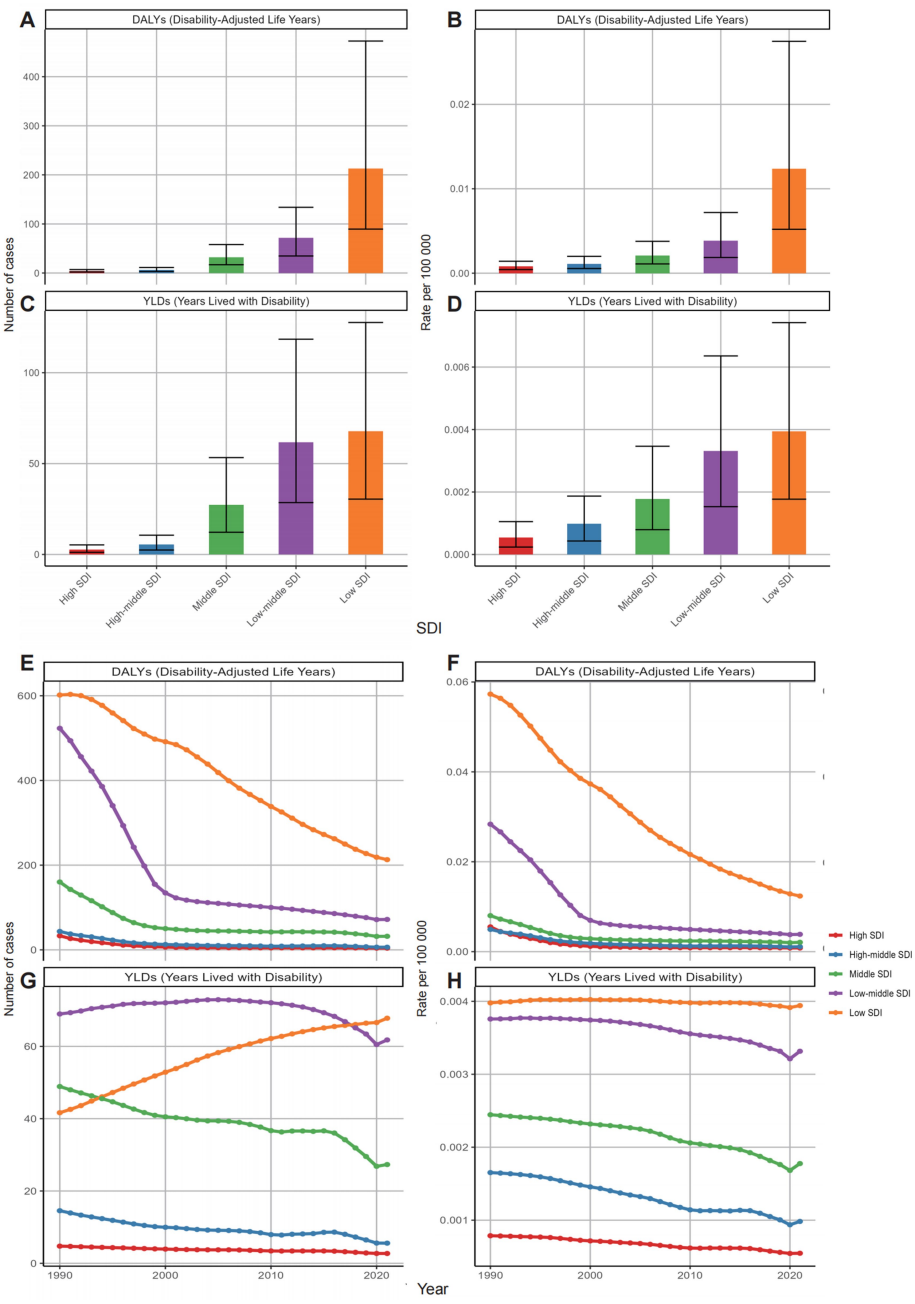
Global distribution (2021) and temporal trends (1990–2021) in neonatal OM attributable to PM2.5 by SDI subgroups: **(A)** DALY cases; **(B)** DALY rates; **(C)** YLD cases; **(D)** YLD rates; **(E)** trends in DALY cases; **(F)** trends in DALY rates; **(G)** trends in YLD cases; **(H)** trends in YLD rates. DALYs, disability-adjusted life years; YLDs, years lived with disability; OM, otitis media; PM2.5, fine particulate matter; SDI, socio-demographic index.

In 2021, DALYs and YLDs decreased with increasing SDI levels. The highest DALY and YLD cases were observed in low SDI regions (Figure 5; Table 1; Supplementary Table S1). DALY cases and rates decreased across all SDI quintiles. The largest DALY rate decrease was observed in low-middle SDI regions (88.7% decrease, EAPC: −5.856). YLD cases in low SDI regions showed a consistent increase, while other SDI regions had moderate declines. YLD rates declined moderately in all SDI regions except in low SDI regions, where they remained stable (Figure 5; Supplementary Table S1).

### 3.4 Decomposition analysis

**Figure 6:**
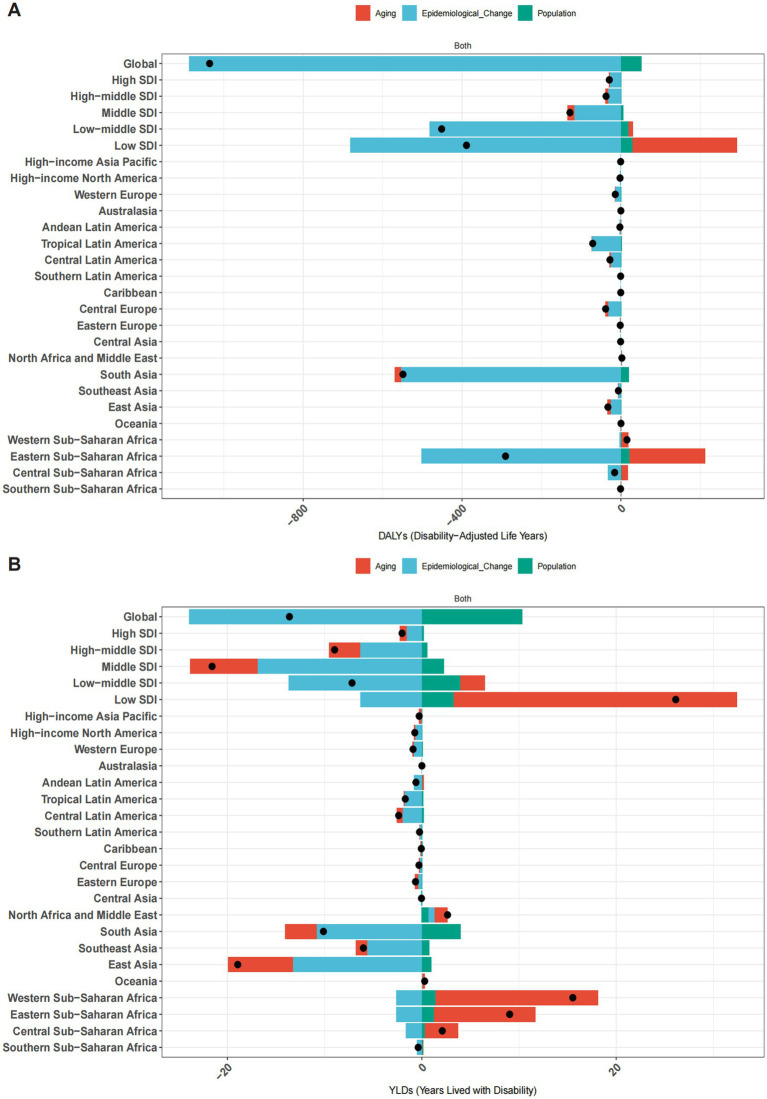
Changes in DALYs **(A)** and YLDs **(B)** for neonatal OM attributable to PM2.5, decomposed by population-level determinants—population growth, aging, and epidemiological changes—from 1990 to 2021 globally, across five SDI regions, and within 21 GBD regions. Black dots represent the total change contributed by all three components. Positive values indicate a contribution to the increase in DALYs or YLDs, whereas negative values indicate a reduction. DALYs, disability-adjusted life years; YLDs, years lived with disability; OM, otitis media; PM2.5, fine particulate matter.

Decomposition analyses indicated that epidemiological changes had a significant impact on the decrease in the burden of neonatal OM attributable to PM2.5 (Figure 6). Globally, epidemiological changes were the primary contributors to this decline in DALYs, accounting for −104.98% of the change, and this pattern was consistent across all SDI regions. Epidemiological changes contributed significantly to the decline in DALYs in low-middle (−106.75%) and low SDI (−175.19%) regions. Notably, aging (67.9%) drove increases in DALYs in low SDI regions. Among the 21 GBD regions, Eastern sub-Saharan Africa (−172.95%) and South Asia (−100.66%) showed the largest epidemiological contribution to the decline in DALYs, while aging (65.48%) contributed to increases in Eastern sub-Saharan Africa (Figure 6A).

For YLDs, epidemiological changes drove declines across all SDI regions, while population growth contributed to increases. Notably, aging (111.52%) was the main driver of rising YLDs in low SDI regions. Among the 21 GBD regions, the decline in YLDs was most significantly attributed to epidemiological factors in South Asia (−106.57%), Southeast Asia (−92.58%), and East Asia (−69.78%). Conversely, the increase in YLDs was most strongly attributable to aging in Central sub-Saharan Africa (−162.99%), Eastern sub-Saharan Africa (−115.57%), and Western sub-Saharan Africa (−107.83%) (Figure 6B).

## 4 Discussion

Our study provides a comprehensive analysis of the global burden of neonatal OM attributable to PM2.5, highlighting a significant decline in DALYs and YLDs over the past three decades. These findings are consistent with broader trends observed in the global burden of disease, where improvements in air quality and public health interventions have contributed to reductions in the disease burden (20). However, our analysis also reveals substantial regional disparities and unique challenges that require targeted interventions.

Epidemiological changes were the primary driver of the decline in the neonatal OM burden attributable to PM2.5. This significant impact underscores the effectiveness of public health interventions aimed at reducing air pollution and improving healthcare infrastructure. For instance, previous studies have shown that improvements in air quality through policy measures have led to substantial reductions in neonatal disorders (20). In China, a combination of air quality improvement measures, hospital-based birth strategies, and enhanced neonatal care has resulted in a significant reduction in the burden of PM2.5-related preterm births (21). These efforts highlight the importance of coordinated public health strategies in mitigating the impact of environmental factors on neonatal health. Regional differences in the burden of neonatal OM attributable to PM2.5 emphasize the need for tailored public health strategies. High-income regions, such as North America and Western Europe, are primarily affected by ambient PM2.5, while low-income regions, such as Eastern sub-Saharan Africa, are more affected by household PM2.5. This distinction suggests that high-income regions should focus on reducing industrial and vehicular emissions, whereas low-income regions should prioritize improving indoor air quality through cleaner cooking fuels and better ventilation (22). These targeted approaches can maximize the effectiveness of public health interventions and optimize resource allocation.

Sex disparities in the burden of neonatal OM are also noteworthy, with higher DALYs observed in male individuals compared to female individuals. This finding is consistent with previous research indicating that male neonates are more vulnerable to adverse health outcomes (23). This sex disparity may be attributed to biological differences, such as higher oxygen requirements and greater susceptibility to oxidative stress in male fetuses (23, 24). In addition, social factors, including differential access to healthcare and care-seeking behaviors, may contribute to this disparity (25). Future research should explore these mechanisms to inform sex-specific interventions.

The increase in YLD cases in low SDI regions, despite a general decline in other regions, is concerning. This trend may be linked to inadequate healthcare infrastructure, limited access to medical care, and higher environmental exposures in these areas (26, 27). These findings highlight the need for targeted interventions to address health inequities and improve healthcare access in low SDI regions. Policies aimed at reducing environmental exposures and enhancing healthcare services in these regions could significantly mitigate the burden of neonatal OM attributable to PM2.5.

It is important to acknowledge the limitations of this study. First, the data relied on estimates from the Global Burden of Disease (GBD) study, which may have inherent uncertainties and limitations in data collection and estimation methods. Second, the analysis assumed a linear relationship between PM2.5 exposure and neonatal OM, which may not fully capture the complexity of the exposure–response relationship. Third, the study did not account for potential confounding factors, such as socioeconomic status and genetic predispositions that could influence the observed trends.

Future research should focus on elucidating the specific mechanisms underlying the observed trends and identifying effective policy interventions to reduce the burden of neonatal OM attributable to PM2.5. Studies should also explore the impact of different sources of PM2.5 exposure, such as household and ambient pollution, on neonatal health outcomes. In addition, research should address the sex-specific vulnerabilities and social determinants of health to inform targeted interventions.

## 5 Conclusion

In conclusion, our study demonstrates a significant decline in the global burden of neonatal OM attributable to PM2.5 from 1990 to 2021, driven primarily by epidemiological changes, which may be largely due to the implementation of effective public health interventions. However, substantial regional disparities persist, with low SDI regions facing higher burdens. Targeted public health interventions, particularly in low-income regions, are essential to further reduce the burden of neonatal OM attributable to PM2.5. Future research should focus on elucidating the specific mechanisms underlying these trends and developing effective policy interventions to address the ongoing challenges in low SDI regions.

## Data Availability

The original contributions presented in the study are included in the article/Supplementary material, further inquiries can be directed to the corresponding author.

## Footnotes

1
https://vizhub.healthdata.org/gbd-results/

2
https://www.healthdata.org/gbd/methods-appendices-2021
