# Supplementary material for: Global, regional, and national burden of neonatal otitis media attributable to PM2.5 air pollution: findings from the Global Burden of Disease study 2021
Source: Front Public Health. 2025 Dec 4;13:1625071. doi: 10.3389/fpubh.2025.1625071 (PMC12711716; doi:10.3389/fpubh.2025.1625071)
Supplement: Supplementary file 1 [file Data_Sheet_1.PDF]

Supplementary Material

Supplementary Table S1 The YLDs cases and rates of neonatal OM attributable to PM2.5 in 1990 and 2021, and EAPC from 1990 to 2021 at the global and regional level.

Location	1990		2021		1990-2021
	**YLDs cases, (95% UI)**	**YLDs rate, per 100, 000 (95% UI)**	**YLDs cases, (95% UI)**	**YLDs rate, per 100, 000 (95% UI)**	**EAPC, %, (95% CI)**
Global	178.925(82.305, 341.825)	0.029(0.013, 0.055)	165.301(75.609, 314.563)	0.025(0.011, 0.048)	-0.493(-0.572, -0.413)
SDI					
High	4.754(2.067, 9.331)	0.008(0.003, 0.015)	2.724(1.187, 5.249)	0.005(0.002, 0.010)	-1.236(-1.331, -1.140)
High-middle	14.549(6.318, 28.067)	0.016(0.007, 0.030)	5.575(2.449, 10.603)	0.008(0.003, 0.015)	-1.913(-2.069, -1.757)
Middle	48.899(22.259, 94.478)	0.024(0.011, 0.047)	27.319(12.220, 53.305)	0.015(0.007, 0.030)	-1.281(-1.407, -1.155)
Low-middle	68.964(32.218, 129.163)	0.040(0.019, 0.074)	61.785(28.528, 118.453)	0.032(0.015, 0.062)	-0.722(-0.783, -0.662)
Low	41.656(19.238, 77.225)	0.046(0.021, 0.085)	67.788(30.437, 127.653)	0.041(0.018, 0.077)	-0.474(-0.511, -0.436)
Regions					
Andean Latin America	1.408(0.549, 2.928)	0.027(0.010, 0.055)	0.804(0.289, 1.816)	0.013(0.005, 0.030)	-2.458(-2.606, -2.309)
Australasia	0.058(0.006, 0.168)	0.004(0.000, 0.011)	0.069(0.009, 0.193)	0.004(0.001, 0.011)	0.133(-0.006, 0.273)
Caribbean	0.978(0.420, 1.985)	0.024(0.010, 0.048)	0.926(0.399, 1.790)	0.024(0.010, 0.046)	0.134(0.047, 0.221)
Central Asia	0.755(0.335, 1.434)	0.008(0.004, 0.015)	0.721(0.326, 1.354)	0.007(0.003, 0.014)	-0.052(-0.346, 0.244)

Central Europe	0.423(0.204, 0.763)	0.005(0.002, 0.008)	0.136(0.062, 0.253)	0.002(0.001, 0.005)	-1.941(-2.216, -1.665)
Central Latin America	4.473(2.049, 8.643)	0.019(0.009, 0.038)	2.090(0.942, 4.075)	0.010(0.005, 0.020)	-2.022(-2.160, -1.883)
Central Sub-Saharan Africa	4.210(1.819, 8.064)	0.041(0.018, 0.078)	6.316(2.671, 12.393)	0.030(0.013, 0.059)	-1.079(-1.143, -1.016)
East Asia	26.534(11.339, 50.857)	0.023(0.010, 0.044)	7.593(3.229, 14.849)	0.009(0.004, 0.019)	-2.046(-2.294, -1.797)
Eastern Europe	0.921(0.432, 1.782)	0.005(0.003, 0.010)	0.276(0.123, 0.532)	0.003(0.001, 0.005)	-2.370(-2.706, -2.033)
Eastern Sub-Saharan Africa	16.212(7.224, 31.023)	0.045(0.020, 0.086)	25.252(11.347, 48.006)	0.040(0.018, 0.075)	-0.494(-0.511, -0.477)
High-income Asia Pacific	0.718(0.284, 1.435)	0.007(0.003, 0.014)	0.444(0.176, 0.915)	0.007(0.003, 0.014)	-0.613(-0.790, -0.437)
High-income North America	1.354(0.560, 2.797)	0.006(0.003, 0.013)	0.633(0.272, 1.278)	0.003(0.001, 0.006)	-2.405(-2.537, -2.273)
North Africa and Middle East	10.234(4.783, 20.062)	0.020(0.009, 0.039)	12.882(5.809, 24.373)	0.021(0.010, 0.040)	0.227(0.108, 0.346)
Oceania	0.329(0.139, 0.638)	0.033(0.014, 0.064)	0.629(0.268, 1.230)	0.033(0.014, 0.064)	0.047(-0.007, 0.101)
South Asia	71.376(33.185, 133.432)	0.045(0.021, 0.085)	61.258(28.229, 117.760)	0.039(0.018, 0.074)	-0.603(-0.668, -0.538)
Southeast Asia	15.960(7.405, 31.090)	0.027(0.013, 0.053)	9.947(4.448, 18.922)	0.018(0.008, 0.034)	-1.485(-1.571, -1.400)
Southern Latin America	0.494(0.180, 1.113)	0.010(0.004, 0.022)	0.263(0.089, 0.598)	0.006(0.002, 0.014)	-1.649(-1.764, -1.534)
Southern Sub-Saharan Africa	2.043(0.869, 4.054)	0.027(0.012, 0.054)	1.675(0.713, 3.366)	0.021(0.009, 0.042)	-0.924(-1.006, -0.841)
Tropical Latin America	3.075(1.386, 6.047)	0.018(0.008, 0.035)	1.375(0.619, 2.646)	0.008(0.004, 0.015)	-2.716(-2.830, -2.602)
Western Europe	1.867(0.803, 3.610)	0.008(0.003, 0.016)	0.967(0.373, 2.053)	0.005(0.002, 0.010)	-1.972(-2.178, -1.766)
Western Sub-Saharan Africa	15.505(7.007, 29.182)	0.043(0.020, 0.082)	31.045(14.366, 59.102)	0.039(0.018, 0.074)	-0.445(-0.496, -0.393)

Abbreviations: YLDs = Years Lived with Disability; OM = otitis media; PM2.5 = fine particulate matter; EAPC = estimated annual percentage change; SDI = socio-demographic index; UI = uncertainty intervals; CI = confidence intervals.
